# Supplementary material for: Transposon insertional mutagenesis of diverse yeast strains suggests coordinated gene essentiality polymorphisms
Source: Nat Commun. 2022 Mar 21;13:1490. doi: 10.1038/s41467-022-29228-1 (PMC8938418; doi:10.1038/s41467-022-29228-1)
Supplement: Supplementary file 1 — Supplementary Information [file 41467_2022_29228_MOESM1_ESM.pdf]

Supplementary materials for

"**Transposon insertional mutagenesis of diverse yeast strains suggests coordinated gene essentiality polymorphisms**" by Chen, Michel, and Zhang (jianzhi@umich.edu)

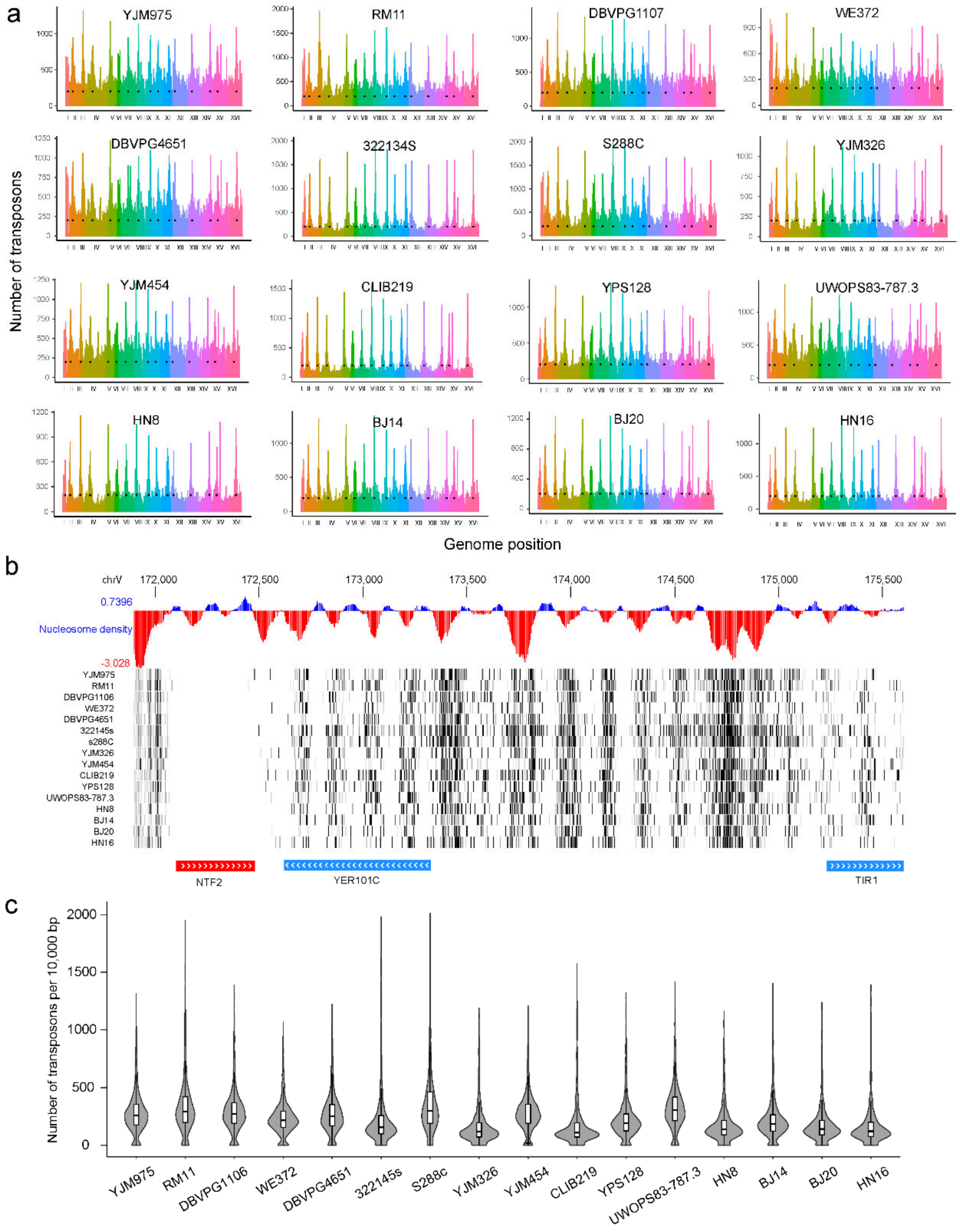

Supplementary Fig. 1. Genome-wide analysis of transposon insertion sites. a, Profile of transposon density across all chromosomes of each strain. Black dots indicate centromeres. Different chromosomes are shown by different colors. Each bar shows the number of transposons in a 10kb region. **b**, Preferential transposon insertions into inter-nucleosomal DNA. Shown here is a typical genomic segment along with the transposon insertions and nucleosomal densities. Each vertical line in the top panel represents the log ratio of the average probe intensity between the nucleosomal DNA and the whole genome DNA at that position. Blue indicates nucleosomal regions while red indicates inter-nucleosomal regions. Each vertical line in the bottom panel represents one transposon insertion. Horizontal bars mark gene locations, with gene names provided below the bars and white arrows indicating transcriptional directions. Gene deletion-based essentiality annotations in S288C are shown by the color of the gene: red for essential and blue for nonessential. **c**, Violin plot of the distribution of the number of transposons per 10kb genomic region (1,208 regions in total) in each strain. In each box plot inside the violin plot, the lower and upper edges of a box represent the first (qu_1) and third (qu_3) quartiles, respectively, the horizontal line inside the box indicates the median (*md*), and the whiskers extend to the most extreme values inside inner fences, $md \pm 1.5(qu_3\text{-}qu_1)$.

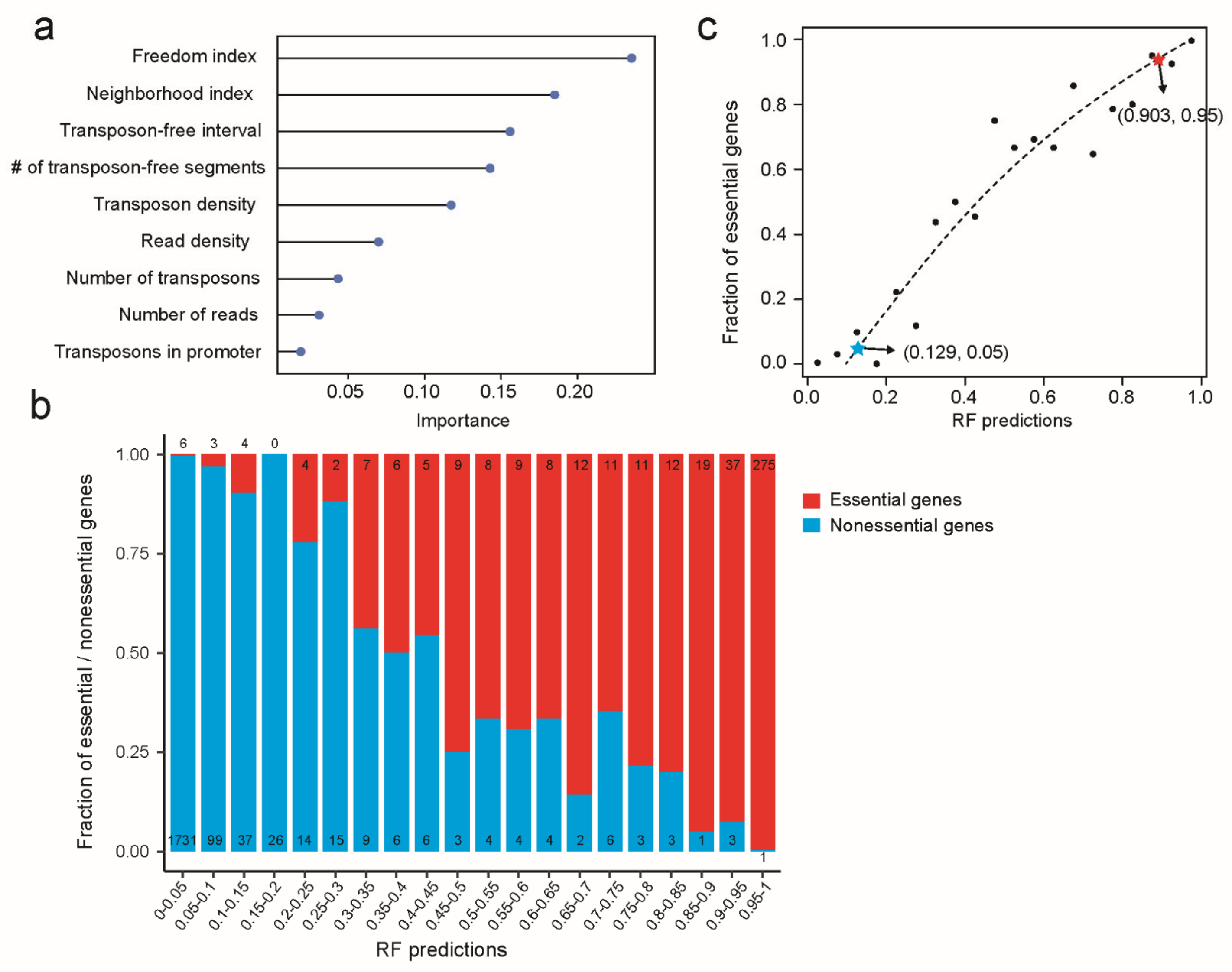

Supplementary Fig. 2. Characteristics and performance of the random forest (RF) classifier of gene essentiality. a, Importance of each feature used in the RF classifier (see Methods for the definition of importance). Features are described in Supplementary Data 3. **b**, Fractions of essential (red) and nonessential (blue) genes in each interval of the RF output. Numbers of essential and nonessential genes are provided at the top and bottom of the bars, respectively. **c**, Polynomial regression between the mean RF output in an interval and the fraction of essential genes in the interval. The thresholds used to identify essential genes and nonessential genes in S288C are marked by a red star and a blue star, respectively.

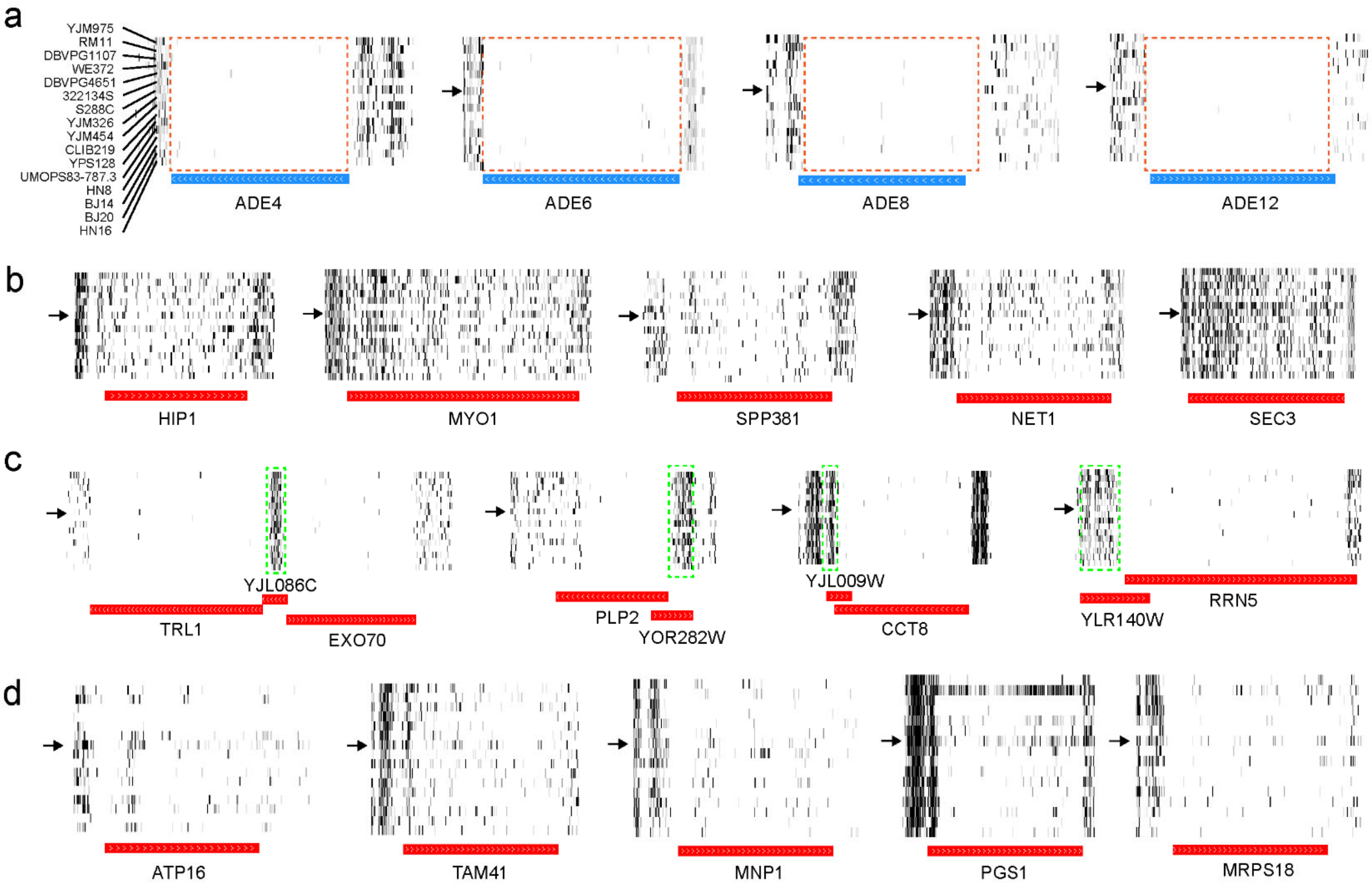

Supplementary Fig. 3. Examples of gene essentiality prediction-annotation disparity. Horizontal bars mark gene locations, with gene names provided below the bars and white arrows indicating transcriptional directions. Gene deletion-based essentiality annotations in S288C are shown by the color of the gene: red for essential and blue for nonessential. **a,** Four adenine biosynthetic genes that are nonessential in the rich medium used in gene deletion studies are essential in the medium used in the present study. **b**, Examples of annotated essential genes that tolerate transposon insertions in the entire coding sequence. **c**, Examples of annotated essential genes that are partially overlapped with other essential genes. The non-overlapping regions that allow multiple transposon insertions are shown in green dashed boxes. **d**, Examples of annotated essential genes that are predicted nonessential because only a small fraction of their coding sequences are transposon-free. In each panel, the black arrow points to S288C.

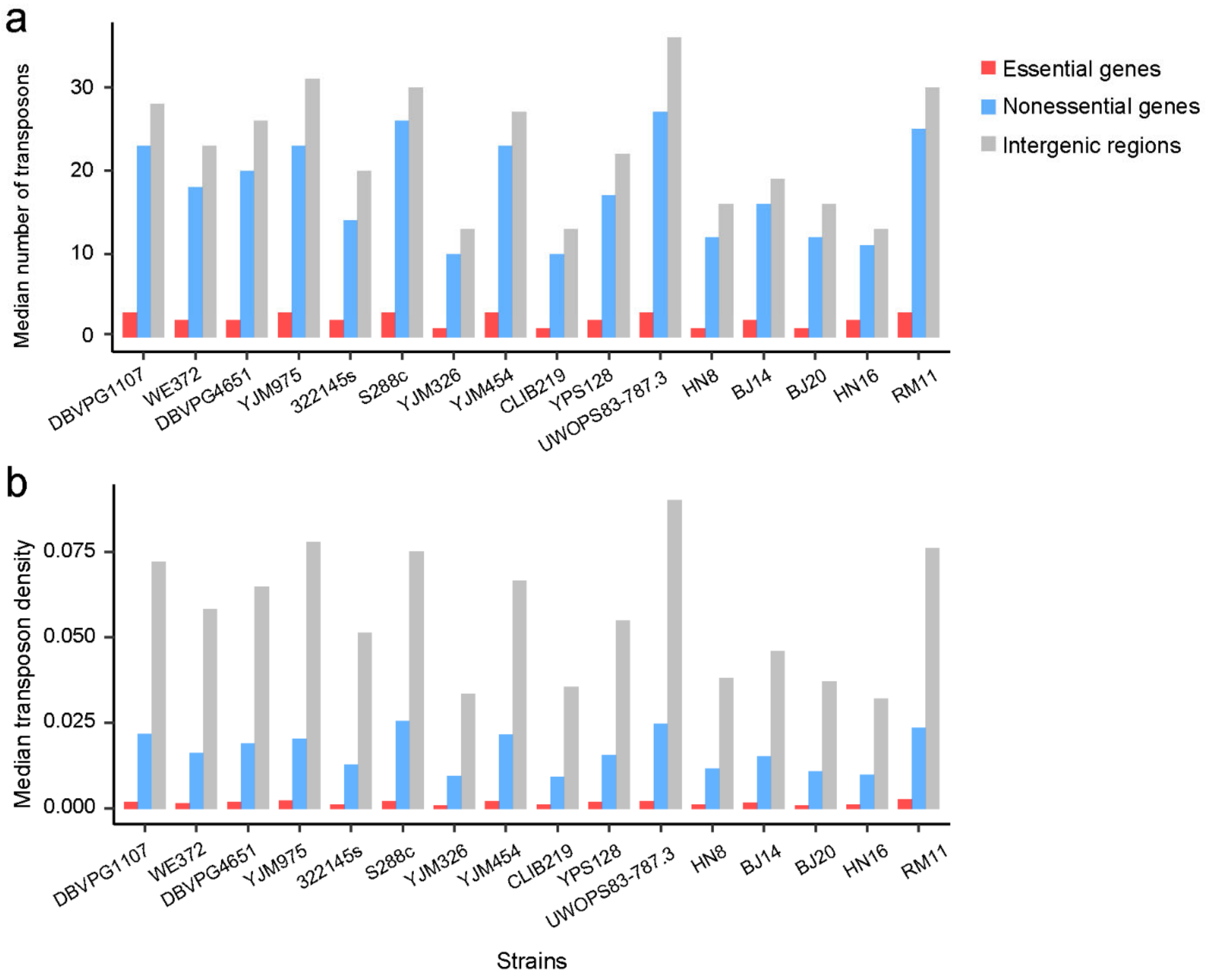

Supplementary Fig. 4. Median number of transposon insertions and median transposon density in each strain in genes with essentiality annotations from S288C and intergenic regions. a, Median number of transposons in each category. **b**, Median transposon density per bp in each category.

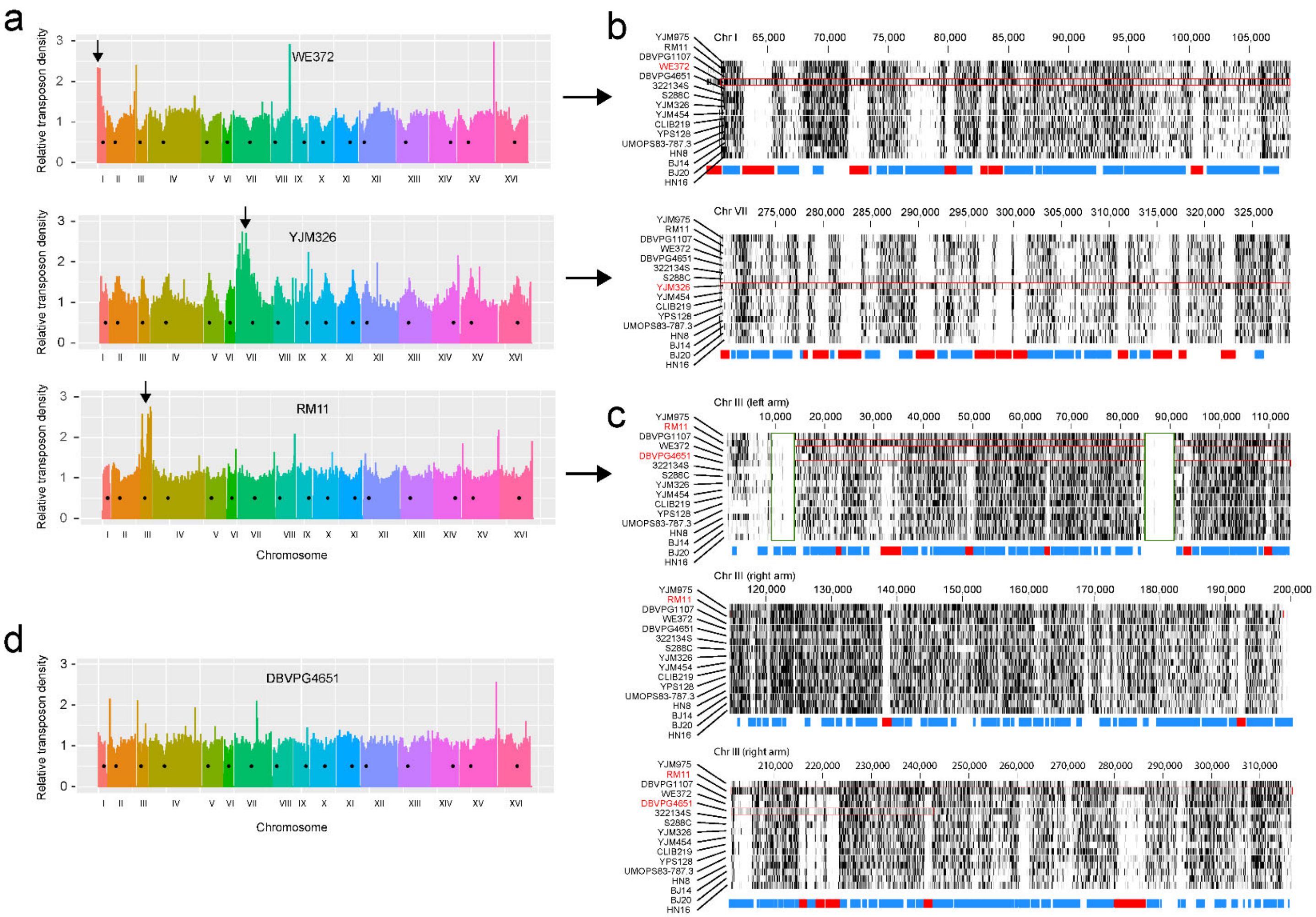

Supplementary Fig. 5. Aneuploidy renders otherwise essential genes tolerant to transposon insertions. a, Relative transposon densities along chromosomes. Y-axis shows the number of transposons in a 10kb window of the focal strain relative to the average number in all other strains for the same window, followed by normalization to a genomic average of 1. Black dots indicate centromeres. Each vertical arrow indicates the suspected presence of an extra copy of a chromosome or chromosomal segment. **b**, The transposon maps for Chromosome I and Chromosome VII across all strains. **c**, The transposon map for Chromosome III across all strains. The top graph is the transposon map of the left arm, whereas the lower two graphs together show the right arm. All essential genes become tolerant to transposon insertions in RM11. The essential genes in the top and bottom graphs but not in the middle graph tolerate transposon insertions in DBVPG4651. **d**, Relative transposon densities along chromosomes of DBVPG4651. In panels b and c, annotated S288C essential and nonessential genes are shown in red and blue, respectively. Red boxes indicate transposon tolerance (potentially) explained by aneuploidy, whereas green boxes are repetitive regions.

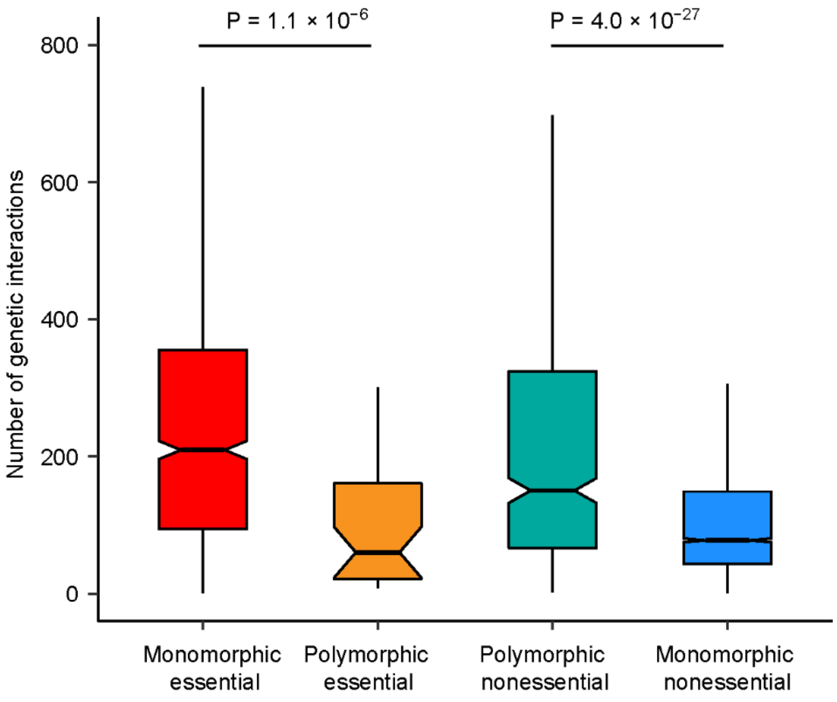

Supplementary Fig. 6. Comparisons of numbers of genetic interactions between monomorphic and polymorphic genes among annotated essential genes and among annotated nonessential genes, respectively. The number of genes in each group (from left to right) is 1046, 38, 529, and 4201. In each box plot, the lower and upper edges of a box represent the first (qu_1) and third (qu_3) quartiles, respectively, the horizontal line inside the box indicates the median (*md*), and the whiskers extend to the most extreme values inside inner fences, $md \pm 1.5(qu_3\text{-}qu_1)$. Notches show the 95% confidence interval of the median. *P*-values are from two-tailed Wilcoxon rank-sum tests. Note that essential genes (the left two bars) should not be compared with nonessential genes (the right two bars) due to the use of different strategies in studying the genetic interactions of essential genes and those of nonessential genes. Specifically, essential genes are typically studied using temperature-sensitive (TS) mutants. Contrasting the deletion of a nonessential gene where the gene activity is completely abolished, TS mutations of an essential gene may only partially reduce the gene activity. The potential remnant activity of the essential gene could lead to an underestimation of its number of genetic interactions. Consequently, the true number of genetic interactions of monomorphic essential genes and that of polymorphic essential genes are likely greater than those observed here. It is therefore inappropriate to compare the number of genetic interactions between polymorphic essential and nonessential genes, or between monomorphic essential and nonessential genes.

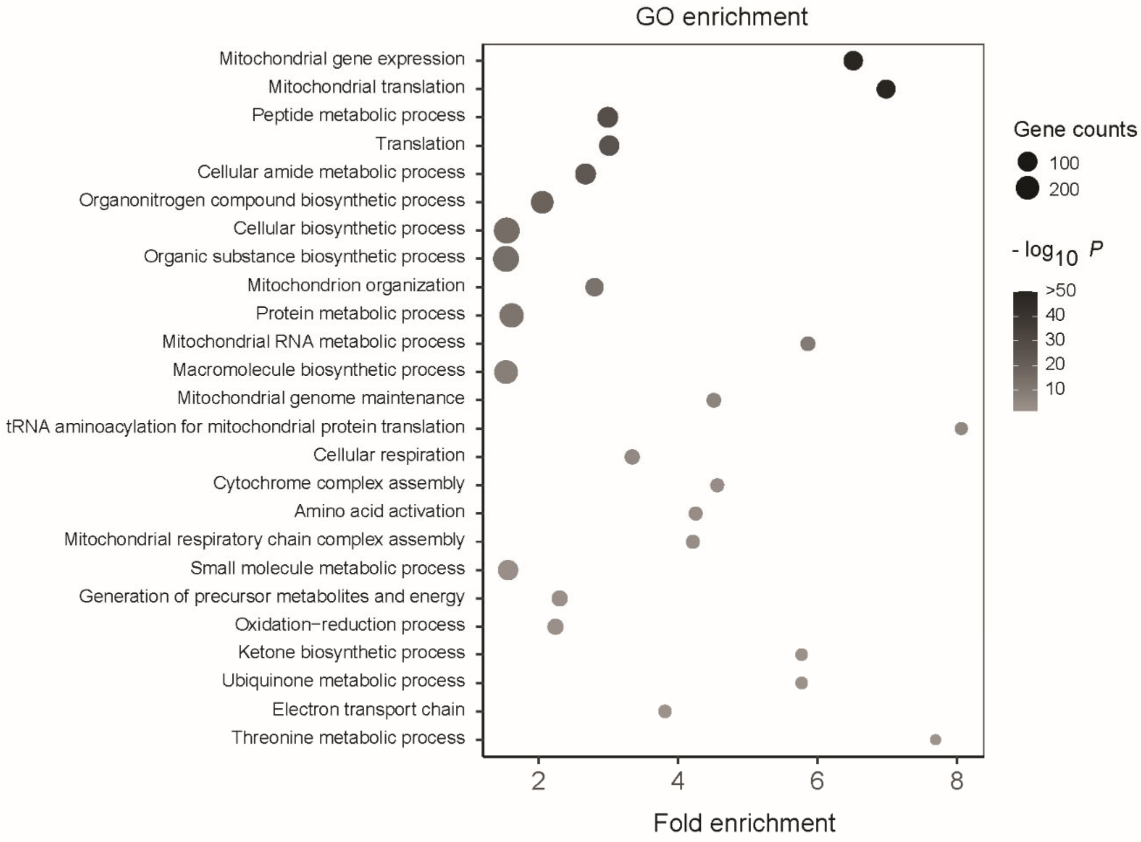

Supplementary Fig. 7. Enrichment of the 567 genes that exhibit essentiality polymorphism in biological process GO terms. Gene count, number of genes in the gene group that belong to the enriched GO term. The shade of a circle indicates the statistical significance of GO enrichment measured by the *P*-value from the hypergeometric test adjusted for multiple testing using the Bonferroni correction.

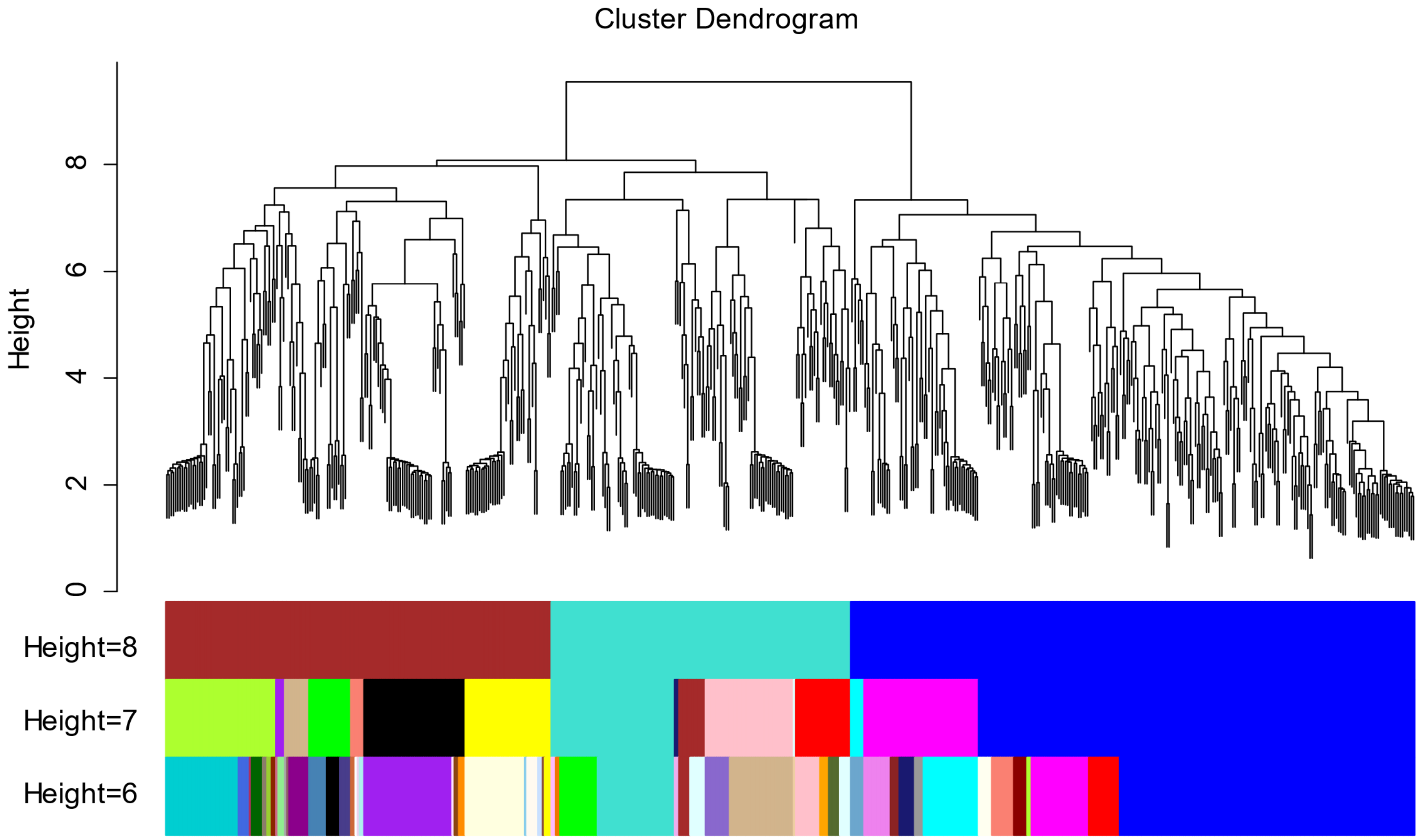

Supplementary Fig. 8. Clustering analysis of the 567 polymorphic essential genes on the basis of the correlation matrix of Fig. 4a. Each tip of the dendrogram represents one of the 567 genes. The color bar below the dendrogram shows gene groups identified from the dendrogram when a cutoff of height = 6, 7, or 8 is applied.
