# Supplementary material for: Transposon insertional mutagenesis of diverse yeast strains suggests coordinated gene essentiality polymorphisms
Source: Nat Commun. 2022 Mar 21;13:1490. doi: 10.1038/s41467-022-29228-1 (PMC8938418; doi:10.1038/s41467-022-29228-1)
Supplement: Supplementary file 3 — Description of Additional Supplementary Files [file 41467_2022_29228_MOESM3_ESM.pdf]

Description of Additional Supplementary Files

File Name: Supplementary Data 1
Description: Overview of the 16 *S. cerevisiae* strains studied

File Name: Supplementary Data 2
Description: Characteristics of the libraries

File Name: Supplementary Data 3
Description: Features used in the Random Forest model for predicting gene essentiality

File Name: Supplementary Data 4
Description: Machine learning features and predictions of gene essentiality in strain S288C

File Name: Supplementary Data 5
Description: Genes annotated as essential because of overlap with essential genes

File Name: Supplementary Data 6
Description: Machine learning features and predictions of gene essentiality from 15 non-S288C strains

File Name: Supplementary Data 7
Description: Genes exhibiting essentiality polymorphism

File Name: Supplementary Data 8
Description: Enrichment in biological process GO terms of the 567 genes exhibiting essentiality polymorphism

File Name: Supplementary Data 9
Description: Correlation between genes in essentiality across strains (with phylogenic relationships considered)

File Name: Supplementary Data 10
Description: Protein complexes including at least two members with gene essentiality polymorphism

File Name: Supplementary Data 11
Description: Metabolic pathways with at least two components exhibiting gene essentiality polymorphism

File Name: Supplementary Data 12
Description: Number of transposons in each coding segment (written in the 5′-to-3′ direction)
